# Crowd Intelligence for the Classification of Fractures and Beyond

**DOI:** 10.1371/journal.pone.0027620

**Published:** 2011-11-23

**Authors:** Joseph Bernstein, Joy S. Long, Christian Veillette, Jaimo Ahn

**Affiliations:** 1 Department of Orthopedic Surgery, Veterans Hospital, Philadelphia, Pennsylvania, United States of America; 2 Department of Orthopedic Surgery, University of Pennsylvania School of Medicine, Philadelphia, Pennsylvania, United States of America; 3 Department of Orthopedic Surgery, University of Toronto, Toronto, Ontario, Canada; Hungarian Academy of Sciences, Hungary

## Abstract

**Background:**

Medical diagnosis, like all products of human cognition, is subject to error. We tested the hypothesis that errors of diagnosis in the realm of fracture classification can be reduced by a consensus (group) diagnosis; and that digital imaging and Internet access makes feasible the compilation of a diagnostic consensus in real time.

**Methods:**

Twelve orthopaedic surgeons were asked to evaluate 20 hip radiographs demonstrating a femoral neck fracture. The surgeons were asked to determine if the fractures were displaced or not. Because no reference standard is available, the maximal accuracy of the diagnosis of displacement can be inferred from inter-observer reliability: if two readers disagree about displacement, one of them must be wrong. That method was employed here. Additionally, virtual reader groups of 3 and 5 individual members were amalgamated, with the response of those groups defined by majority vote. The purpose of this step was to see if increasing the number of readers would improve accuracy. In a second experiment, to study the feasibility of amassing a reader group on the Internet in real time, 40 volunteers were sent 10 periodic email requests to answer questions and their response times were assessed.

**Results:**

The mean kappa coefficient for individual inter-observer reliability for the diagnosis of displacement was 0.69, comparable to prior published values. For 3-member virtual reader groups, inter-observer reliability was 0.77; and for 5-member groups, it was 0.80. In the experiment studying the feasibility of amassing a reader group in real time, the mean response time was 594 minutes. For all cases, a 9-member group (theoretically 99% accurate) was amassed in 135.8 minutes or less.

**Conclusions:**

Consensus may improve diagnosis. Amassing a group for this purpose on the Internet is feasible.

## Introduction

The classification of femoral neck fractures proposed by Garden [1] in 1961 has gained widespread use. Indeed, the basic feature of this classification— namely, the presence or absence of displacement—is said to determine treatment: among elderly patients with comparable medical histories, fractures which are defined as non-displaced are said to need pinning, whereas for patients with displaced fractures, joint replacement is recommended [2].

The detection of displacement in the case of femoral neck fractures is a task for which surgeons express great confidence. A prior report [3] surveyed members of Orthopaedic Trauma Association and surgeons at European clinics affiliated with AO International and found that 96% of the surgeons felt they could differentiate between non-displaced (Garden Types I and II) and displaced (Types III and IV) fractures. Despite that confidence, it may be the case that orthopaedic surgeons are not able to recognize the presence or absence of displacement on radiographs with high accuracy.

Deficits regarding diagnostic accuracy may escape easy detection. That's because a direct observation reference standard is not available: femoral neck fracture classified as non-displaced are typically not opened surgically; and those classified as displaced are subjected to arthroplasty, a treatment that could displace a fracture that was not displaced pre-operatively. Accordingly, there are to our knowledge no studies explicitly assessing the accuracy of radiographic diagnosis of femoral neck fracture displacement. Nevertheless, poor radiographic accuracy can be inferred from prior studies in the medical literature.

The first clue comes from the study of Totterman et al [4]. In their study, five orthopaedic surgeons were asked to measure displacement in 10 cases of femoral neck fractures as seen on plain radiographs. They were asked to interpret radiographs twice, with an interval of 3 months between viewings. They found that individual readers disagreed with their own assessment by more than 10.7 mm on average (recalculated from table 1 in their report [4]). The mean displacement of all ten cases was 15.9 mm, but the range of values (lowest to highest measurement) was, on average 20.7 mm.

**Table 1:** A demonstration of the consensus classification of virtual reader groups, as a function of individual classifications on a sample case.

INDIVIDUAL READER	Stated Classification
Reader-1	Displaced
Reader-2	Non-displaced
Reader-3	Non-displaced
Reader-4	Displaced
Reader-5	Displaced

SAMPLES of VIRTUAL READER GROUPS	Derived Classification
Group comprising Reader-1, Reader-2, Reader-3. Reader-4 and Reader-5	Displaced
Group comprising Reader-1, Reader-2, Reader-3	Non-Displaced
Group comprising Reader-2, Reader-3, Reader-4	Non-Displaced
Group comprising Reader-3, Reader-4, Reader-5	Displaced

Another suggestion about limited accuracy comes from the femoral neck fracture displacement studies of Oakes et al [5] and Thomsen et al [6], both of which reported mean kappa coefficients for inter-observer reliability in the range of 70% (0.73 and 0.68 respectively). Inter-observer reliability in the range of 70% for the presence or absence of displacement implies that the maximal average accuracy of reader is approximately 85%. That is because when reader-A says “displaced” and reader-B says “non-displaced” one of them must be wrong. And if there is agreement in only 70% of cases, then there are at least 30 incorrect responses for every 200 observations (as each paired comparison represents two total observations), ie maximal average accuracy is 85%. It is of course possible that average accuracy is even lower than 85%, as readers can agree and it is the case that both are wrong. A binary diagnosis agreement rate of 0.81—a level deemed by conventional standards [7] to be “excellent”—implies that one out of ten cases is misdiagnosed.

Accordingly, the aims of this study are three:

- first, to confirm or refute the work of Oakes et al and Thomsen et al;
- second, assuming that a low mean coefficient of reliability will be found, to test the use of consensus diagnosis to increase accuracy; and
- third, assuming that consensus diagnosis can improve accuracy, to test the feasibility of compiling a consensus in real time.

Consensus diagnosis is based on a phenomenon described by Francis Galton [8]. In 1907, Galton observed that in a country fair contest, the weight of an ox was estimated poorly by individuals, yet the mean of these guesses was within 99.2% of the true value—a collective estimate that was more accurate than the estimates given by cattle experts. From that observation, it was recognized that the aggregation of information from groups might yield better decisions and solutions than could have been offered by individual experts. The expertise possessed by the group *in toto* has been termed “the wisdom of the crowd”[9] or “crowd intelligence”.

Our study, accordingly, comprised two sequential investigations. First, we repeated a traditional experiment of reliability applied to a modified Garden [1] classification, but extended the analysis by aggregating individual readers into three and five member “virtual reader groups”, to see if these groups could detect displacement with greater reliability. In the second phase of the study, we assessed the feasibility of gathering a consensus in real-time by measuring the response time of volunteer orthopaedic surgeons who were sent periodic emails inviting them to interpret an image posted on the Internet.

Together, these investigations not only outline the classification-by-consensus approach but foreshadow the application of crowd intelligence for error reduction in medical practice in general.

## Methods

### Ethics statement

This study was approved by the Philadelphia Veterans Hospital IRB, protocol #01165. There were no patients in the study; rather we used anonymous de-identified radiographs culled from the department's files, long after the patient who xrays was used had been treated. The physician evaluators provided verbal consent to participate, a method sanctioned by IRB protocols.

### Traditional reliability experiment

A sample of radiographs from 15 patients 65 years of age or older who were treated surgically for femoral neck fractures and for which pre-operative AP and lateral x-rays were available was assembled from our department's records. The films of the first five cases were duplicated (for the assessment of intra-observer reliability) yielding a set of 20. The cases were reviewed by 12 orthopedic surgeons. These readers were of three types: six attending arthroplasty surgeons; four attending orthopedic traumatologists; and two orthopedic residents. Of the arthroplasty surgeons, five devoted their practice to joint reconstruction. The sixth reader had a more general practice, but had performed more than 2000 hip surgeries at the time of the study. Of the four readers who were designated to be traumatologists, three had full time practices dedicated to adult orthopedic trauma; one completed a trauma fellowship, but his practice now focused on hand surgery. The two orthopedic residents were in their second and fifth year, respectively, at the time of the study session.

Each reader was instructed to classify the fracture pattern, using the scheme proposed by Garden. To assess intra-observer reliability, the first five x-rays were duplicated and shown a second time at the end of the session.

The four category Garden classification was, for the purpose of analysis, compressed into a modified two-category scheme: types I and II were considered to be modified type A (broadly, “non-displaced”) and types III and IV were considered to be modified type B (“displaced”).

For every case, the individual assessments of all readers were compared pair-wise to assess the inter-observer reliability. The responses for the set of five repeated cases were then also compared for each individual reader, to determine the intra-observer reliability.

All rates of agreement were given as modified kappa coefficients[10]—ie, the rate of agreement adjusted for chance—as given by the following equation *K* = (P_0_ −P_c_)/(1−P_c_), where P_0_ is the observed agreement rate and P_c_ is the chance agreement rate.

Iterating through the data matrix, the readers' responses were aggregated to determine the modal response; and the responses of each reader were then compared to the mode for each case.

### Virtual reader group experiment

To study the effect of consensus, virtual reader groups (VRGs) were created from the pool of respondents. First, the 12 readers were arbitrarily segregated into two sets: the six arthroplasty surgeons in one, with the four attending traumatologists and the two orthopedic residents in the other. From both sets of six surgeons, all 20 possible groupings of three readers were specified, e.g., VRG-1 = “Dr-1, Dr-2, Dr-3”; VRG-2 = “Dr-1, Dr-2, Dr-4”; VRG-3 = “Dr-1, Dr-2, Dr-5”, etc. The consensus diagnosis for displacement was recorded by simple majority (Table 1). The inter-group reliability coefficient was then assessed, comparing each of the 20 arthroplasty VRGs to each of the 20 non-arthroplasty VRGs, 400 pairs in all.

The decision was made *a priori* to compare only arthroplasty VRGs to non-arthroplasty VRGs, and not every possible triplet, for two reasons. First, assessing all possible triplets would have been unwieldy as there are 220 possible 3-member groups that can be selected from a set of 12, yielding 48,180 possible pairs. Second, many of these groups would have shared a majority of members, rendering a comparison self-referential and uninformative.

The reliability analysis was then repeated for the twelve possible five-member VRGs.

### Email response time experiment

Forty attending orthopaedic surgeon were recruited from the national academic community as unpaid volunteers. None of these surgeons participated in the first phase, but that was not a deliberate consideration. Each of these surgeons provided an email address and consent to receive periodic solicitations. Ten times over a period of one month, approximately once every three days, these surgeons were sent an email request to visit a web page and answer a question based on an image. The volunteers were informed that the only datum collected was the response time.

For each case, the number of surgeons who responded within three days was counted, with those who responded after that point or not at all designated as non-responders. By assessing the difference between the time of response and the time of notification, the individual response delay time was calculated. The mean delay time for the group of responders was calculated for each case, and for all ten cases over all.

Using an accuracy rate suggested by the data (below) of 82%, it was mathematically determined that to create a group with an aggregate accuracy rate of 95% five members would be needed; similarly, a nine-member group would be 99% accurate. (That is to say, if each individual is likely to offer a correct answer 82% of the time, a five member group will likely have 3 more members offering a correct answer 95% of the time and a 9 member group will have 5 more members offering a correct answer 99% of the time.) As such, we collected response-time data to determine how long it would take to amass groups with at least five or nine responders.

## Results

### Traditional reliability experiment

The mean kappa coefficient for intra-observer reliability was 0.8 (see Table 2). The mean kappa coefficient for inter-observer reliability was 0.69. The mean rate of agreement with the modal response was 0.82. The consensus classification was often strongly defined: in 16 of the 20 cases, the distribution of votes was at least 10–2.

**Table 2:** Performance by reader assessing displacement.

Reader	Intra-observer Reliability	Inter-observer Reliability	Agreement Rate with Consensus
Arthroplasty Attending-1	1.00	0.71	0.90
Arthroplasty Attending-2	0.60	0.76	1.00
Arthroplasty Attending-3	0.60	0.80	1.00
Arthroplasty Attending-4	0.60	0.69	0.80
Arthroplasty Attending-5	0.60	0.71	0.80
Arthroplasty Attending-6	0.60	0.80	1.00
Resident-1	0.60	0.33	0.40
Resident-2	1.00	0.52	0.70
Trauma Attending-1	1.00	0.71	0.80
Trauma Attending-2	1.00	0.80	0.80
Trauma Attending-3	1.00	0.71	0.80
Trauma Attending-4	1.00	0.75	0.80
Mean, Entire Group	0.80	0.69	0.82
Arthroplasty Attending Sub-group Mean	0.67	0.75	0.92
Resident Sub-group Mean	0.80	0.43	0.55
Trauma Attending Sub-group Mean	1.00	0.74	0.80
Attendings only Sub-group Mean	0.80	0.74	0.87

### Virtual reader group experiment

The mean reliability for classification by 3-member virtual reader groups was 0.77. For the 5-member groups, the mean reliability was 0.80.

### Email response time experiment

On average, 35 out of the 40 orthopaedic surgeons responded within three days of solicitation, with a mean response time among responders of 594 minutes, or approximately 10 hours (see Table 3). The average delay between solicitation an attainment of groups of size 5 or 9 was 19.4 and 55.4 minutes, respectively. The single largest time interval needed to collect 9 responses was 135.8 minutes, meaning that for all ten cases, a group of 9 was assembled in less than 2.5 hours.

Of the 40 orthopaedic surgeons, 17 replied to all 10 cases within 3 days; 12 replied to 9 and 6 replied to 8; that is, 35 replied to 8 or more cases.

**Table 3:** Response time to 10 solicitations.

Case	Day of the week case was sent	Number of responders within three days	Mean response time in minutes among responders	Minutes needed to build a group of 5 responders	Minutes needed to build a group of 9 responders
1	Saturday	31	1287	23.7	135.8
2	Tuesday	38	329	5.6	24
3	Friday	36	731	5.2	17.7
4	Monday	37	397	34.6	65.2
5	Thursday	35	428	14.3	34.2
6	Sunday	35	498	27.4	58.6
7	Wednesday	29	548	17.9	35.9
8	Saturday	34	737	37.7	121.6
9	Tuesday	37	365	18.4	59.5
10	Friday	36	617	22.1	49.4
MEAN FOR	ALL CASES	34.8	594	19.4	55.4

## Discussion

In previously reported studies, fracture classification systems of apparent merit were found to lack the reliability necessary for clinical use [11]. In the present study, we investigate the possibility that the application of a classification may be hindered by human error, and that a consensus classification approach may improve reliability.

We performed a traditional experiment of reliability on a modified Garden classification. We reported a mean kappa coefficient for inter-observer reliability of 0.69, comparable to the 0.73 value found by Oakes et al [5] and the 0.68 value determined by Thomsen et al [6].

We also found that the mean rate of agreement with the consensus was 82%. This corresponds to the accuracy rate implied by the observed 0.69 kappa coefficient, as accuracy is approximated by the square root of kappa. The concordance between the consensus-agreement rate, 82%, with the square root of kappa, 0.83, suggests that in cases lacking a reference standard, consensus-agreement rates can be used as a proxy for individual accuracy rates.

In the second step of analysis, we formed virtual reader groups, to assess the effect of consensus classification. These virtual reader groups were indeed able to classify hip fractures with greater reliability: contrasted with the individual inter-observer reliability of 0.69, the mean reliability was 0.77 for classification by 3-member virtual reader groups and 0.80 for 5-member groups.

To test the feasibility of assembling groups of readers in real time, we timed the response of 40 volunteers to email queries for image interpretation. We found that a group of 9 (one theoretically 99% accurate) could be assembled in three hours, even on the weekend.

Limitations of our study must be considered. To begin, the effect of group reading was studied apart from the study of feasibility. That split reflects the chronology of discovery: first, the improved accuracy of group reading was detected, and only later, to address the issue of practicality, was a determination of response times undertaken.

Furthermore, it may seem that the results presented are obvious; that it should be apparent that having more readers leads to greater accuracy. That is not always true: increasing the number of readers only enhances the proclivities of the individual reader. If individual accuracy was less than 50%, increasing the number of readers would indeed decrease accuracy. Thus, one must demonstrate that the individual reader accuracy exceeds 50%. A second necessary finding is that no particular case was particularly difficult. In the instance where overall accuracy is, say, 80%, yet that rate is based on an accuracy of 100% in the 80% of cases which are "easy" and 0% accuracy in the remaining 20% of cases which are "hard", increasing the number of readers will not improve things: the hard cases will continue to vex the readers.

It must also be considered that our email response time experiment represents a “best case scenario”: the task was easy and of low stakes, and a series of ten may have been too short to evoke fatigue, apathy or other causes of waning interest. That said, the study population was small and perhaps employing a larger group may more than compensate for the inevitable drop-outs. Additionally, if group members were to be reciprocally rewarded, so to speak –by having their own cases read by their peers- attrition may be less of a concern.

Two general criticisms of fracture classification studies such as ours apply here: first, that the volunteer reviewers simply do not care as much as attending surgeons and therefore devote less mental effort to the task of diagnosis and second, that the cases were not representative of the true distribution seen in clinical practice (a form of spectrum bias). These cannot be answered beyond the equally general reply, namely, that this is a feature of all studies of this type.

### Conclusion

In sum, we have found that harvesting the wisdom of the crowd may help improve fracture classification reliability, suggesting that group efforts might improve diagnostic accuracy in general. This is consistent with the experimental behavioral investigations reported in *Science* by Wooley et al[12] who found “converging evidence of a general collective intelligence factor that explains a group's performance on a wide variety of tasks.” Of course, not all crowds are wise: crowds can be susceptible to “madness” and “extraordinary delusions” [13]. To create a wise crowd, we need to have diversity of opinion; we need to ensure that opinions are based on some form of knowledge; and we must make certain that an individual's opinions remain independent of others' opinions. Those criteria can be met in the case of fracture classification, and perhaps other clinical problems in orthopaedic surgery and medicine. Thhe advice of a wise crowd can be used to supplement (and not supplant) our individual powers of reason. In turn, crowd intelligence may help us reduce error and improve the quality of care at low additional cost.
